# Posttraumatic Growth and Related Factors of Child Protective Service Workers

**DOI:** 10.1186/2052-4374-25-6

**Published:** 2013-05-21

**Authors:** Young Sun Rhee, Young Bin Ko, In Young Han

**Affiliations:** 1 Department of Administration and Social Welfare, Chosun University, Kwangju, South Korea; 2 Department of Social welfare, Ewha womans University, Seoul, South korea

**Keywords:** Posttraumatic growth, Vicarious trauma, Coping strategy, Social support

## Abstract

**Objectives:**

The aim of the study is to measure the level of vicarious trauma, posttraumatic growth (PTG), and other factors affecting PTG among child protective service workers.

**Methods:**

We include posttraumatic stress, social support, stress coping, and demographic data as independent variables. Data was collected from 255 full-time social workers from 43 child protective agencies as acomplete enumeration and 204 included in the final analysis.

**Results:**

The major findings of the study were as follows: The mean score of PTG was 44.09 (SD:21.73). Hierarchical multiple regression was adopted and "pursuing social support as a way of coping with stress" was the strongest predictive factor (β=0.319, p<0.001) of PTG.

**Conclusion:**

We suggest that child protective workers are vulnerable to posttraumatic stress and mental health services are indicated. We also recommend various types of training for stress coping program, especially strengthening the social support system of the child protective service workers in South Korea.

## Introduction

Child abuse is a traumatic experience that has long term and serious effects on abused children [1]. Incident like child abuse have repercussions for the whole of society, not just the abused persons. People who have experienced these kinds of incident may develop in positive or negative ways. They may feel the similar rage against perpetrator with abused persons, their philosophy about child rearing or their view of life may change, or their relationships with others can become closer or more distant. With regard to the stress that direct victims of child abuse suffer, measures have been put in place by agencies for protecting children. However, the effects of child abuse on persons who indirectly experience it have attracted little attention. Social workers who work in child protective agencies, in particular, receive reports on such cases and investigate related sites, provide services for parents and victims, and conduct work to prevent child abuse. In the process of providing direct and close support to the abused children and their parents, including counseling [2], they inevitably come to have knowledge about these kinds of incidents, even if they do not have direct experience with the traumatic incidents. Through such empathic relations, they come to understand the experience of clients and can vicariously experience the trauma. In the case of trauma experienced vicariously by counselors, they can sometimes experience the symptoms of those who have experienced direct trauma, such as intrusive thoughts or avoidant reactions, sleepless states, and emotional stress [2-6]. In addition, according to some studies, counselors may experience changes in their desires, trust in themselves and others, interpersonal relations, perceptions and memories, identity or view of the world [7]. According to studies on the vicariously experienced trauma of fire fighters and police officers, there are many cases where post-traumatic stress has been reported in relation to cases where children are the victims [8]. In this way, counselors talking with abused children undergo significant stress. The psychological and social health of social workers in child protective agencies is directly related to the quality of child protective services, and social workers often mediated changes of abused children, their parents, and aggressors, so it should be a priority to protect them psychologically. Therefore, it is necessary to understand the trauma experienced vicariously by social workers and to strengthen protective measures to protect them and to enable them to carry on with their jobs.

A traumatic experience becomes an opportunity not only to experience negative emotions but also can bring about positive changes in the personal lives of those who have suffered [9,10]. Growth after suffering trauma can be manifested as increases in confidence resulting from coping with seriously stressful incidents, increases in social resources, increases in personal resources like management techniques, changes in spiritual beliefs, adjustment of one’s life priorities, gratitude for life, and changes in one’s philosophy of life [9]. According to previous report, growth after trauma can take place when people experience trauma vicariously as well as experiencing it directly [11]. Counselors dealing with sexual violence [12], paramedics [13,14], nurses [14,15], and social workers [16] are indirectly exposed to traumatic incidents and experience growth after such trauma by experiencing the trauma vicariously. Although these professionals suffer from stress due to their jobs, growth after trauma can be especially important in preventing exhaustion and job turnover [13,17].

Efforts should be made to develop interventions to facilitate the growth of social workers of child protective agencies who inevitably experience ongoing vicarious trauma. In the Republic of Korea, several studies have been conducted about trauma that is vicariously experienced because of one’s job [2,4-6,12], but such efforts are not enough. Regarding growth after trauma, Gwon and Kim (2006) discussed the growth psychotherapists experience as a result of vicarious trauma. Studies conducted outside Korea have indicated that social support, religion, gender, age, coping strategies, and post-traumatic stress are factors related to growth after vicarious trauma [13,18,19], but factors related to growth after trauma for social workers at child protective agencies in Korea are expected to be different in kind and degree due to the unique aspects of Korean culture.

This study seeks to examine factors related to growth after trauma by considering its general features, the effects of vicarious trauma reported in existing studies, social support, variables regarding stress coping, and workplace conditions of social workers at child protective agencies. To this end, a survey of social workers at child protective agencies in Korea was conducted to understand the social workers’ post-traumatic growth.

## Materials and methods

### Study subjects and study design

For the study, a survey was conducted with 255 child protective service workers, as complete enumeration. They were from the nation’s 43 child protective agencies who perform case management and a cross-sectional study design was used. Data were collected through the mail for two weeks from April 21 to May 5, 2008. For the final analysis, 204 questionnaires were used, with 11 excluded for incomplete responses.

### Study tool

#### Growth after trauma

The Posttraumatic Growth Inventory (PTGI) was used to measure growth after trauma. This tool was developed by Tedeschi and Calhoun (1996), [11] and Lee (2009) [20] Validated it . This 21-item instrument is divided into five subscales, including seven items about relating to others (changes in the meaning of interpersonal relationships), five about possibilities (discovering new possibilities), two about spiritual matters (changes in spiritual interest), five about personal strength (identifying individual strengths) and three about appreciation of life (appreciation of life). Respondents replied according to a six-point Likert scale, from “no change” (0 points) to “remarkable change” (five points): more points meant greater growth. According to an earlier study [20], the Cronbach's α coefficient was 0.95. In this study, it was 0.952.

#### Factors related to vicarious trauma

For the variables related to vicarious trauma, the most shocking child abuse incidents, the level of psychological pain at the time of the incidents, and scores related to the stress felt after vicarious trauma were measured. Respondents were asked which child abuse incident was most shocking and they were required to give answers about the level of psychological pain resulting from that incident [9,11], from 1 point (not painful at all) to 10 points (the most serious imaginable pain). The variable of the level of psychological pain was included in the study design on the assumption that growth after trauma appeared as a result of cognitive processing which took place to handle the psychological pain after vicarious trauma. Regarding posttraumatic stress, the revised Korean version of the Impact of Event Scale (IES-R-K), which was originally developed by Horowitz (1979) [21], revised by Weiss et al. (1997) [22], and adapted by Eun et al. for Korea (2005) [23] was used. Eun also confirmed the reliability and validity of the IES-R-K. The IES-R-K consists of 22 items on 3 subscales: hyper-arousal (6 items), evasion (8 items), and invasion (8 items). Responses are collected on a 5-point Likert scale, from “not at all” (0 points) to “very frequently” (4 points). In addition, groups of stress disorder after trauma can be classified on the threshold of 24/25 points, the level of the cut-off value for PTSD selection. In Eun’s study [23], the Cronbach's α coefficient was 0.87. In this study, the coefficient was 0.964.

#### Factors related to social support

Regarding social support-related factors, respondents were asked to give answers about the following questions. How much support have agencies provided in crises? Have you sought support from them? Have you obtained counseling from them? Additionally, a question asking if they were satisfied with current relations with colleagues and supervisors was included in the questionnaire. The level of support in crises was measured using a Korean version of Crisis Support Scale (CSS) originally developed by Elklit et al. (2001) [24]. A Korean living outside Korea with a good command of English and Korean and a cultural anthropologist participated in the translation job and the Korean version of the scale was developed through meetings of researchers. The CSS consists of 7 questions asking 1) if there were persons who would listen to stories in crises, 2) if respondents could share their stories with persons with similar experiences, 3) if respondents could speak about their thoughts and feelings, 4) if respondents were understood by other persons, 5) if respondents received substantial help from other persons, 6) if respondents were disappointed with persons whom they believed would be supportive, and 7) if respondents were generally satisfied with the support that they received. A 7-point Likert scale was used, from “not at all” (1 point) to “always so” (7 points). A higher number of points means higher social support. In Elklit’s study [24], the Cronbach's α coefficient was. In this study, the coefficient was 0.812.

#### Methods for coping with stress

Also used was the Coping Strategy Indicator (CSI), which was developed by Amirkhan (1990) [25] and whose adequacy was verified by Shin and Kim (2002) [26]. The scale for evaluates the ability to cope with stress positively and which ways of coping with stress have been most frequently used. This 33-item scale was composed of 3 subscales: “ways to pursue social support” (11 items) on coping with stress through seeking advice or information, “problem solving-centered methods” (11 items) on directly solving problems through confronting them instead of avoiding them, and “avoidance-centered coping methods” (11 items) on avoiding problems instead of directly solving them. A 3-point Likert scale was used from “not used at all” (1 point) to “frequently used” (3 points). More points meant that they were more active in coping with stress. In Shin and Kim’s study [26], the Cronbach's α coefficient was 0.84. In this study, it was 0.912.

#### Sociodemographic characteristics

Data on the sociodemographic variables of gender, age, educational background, religion, and marital status were collected.

#### Data analysis methods

General characteristics and basic statistical data were obtained through descriptive statistics, and Cronbach's αwas found through reliability analysis about each scale. The *t*-test, one-way analysis of variance (ANOVA) and regression analysis were conducted for univariate analysis to identify factors affecting posttraumatic growth (PTG). A model was formed with variables found to be significant in univariate analysis and independent variables were classified into four groups: sociodemographic factors, vicarious trauma-related factors, social support factors and stress coping factors. Then, hierarchical linear modeling was conducted. Multi-collinearity was taken into consideration for forming the model.

## Results

### General properties of respondents

About 59.3%, or 121, of the respondents were women. About 58.8%, or 120 persons, were in their 20s and 35.8%, or 73 persons, were in their 30s. A majority of the social workers, 179 or 88.2%, were graduates of colleges or universities that provided a national licensure for the job and 24, or 11.7%, had completed graduate school. About 82.3%, or 167 persons, had religious beliefs and 71.6%, or 146 persons, were single (Table 1).

**Table 1:** **Sociodemographic characteristics of social workers** (**n**=204^*^)

**Characteristics**		**Number**	**(%)**	**Mean**	**S**/**D**
Sex (n=204)	Male	83	(40.7)	-	-
	Female	121	(59.3)	-	-
Age (n=204)	20s	120	(58.8)	-	-
	30s	73	(35.8)	-	-
	40s	11	(5.4)	-	-
Education (Graduate) (n=203)	University	179	(88.2)	-	-
	Graduate school	24	(11.8)	-	-
Religion (n=203)	Religious^†^	167	(82.3)	-	-
Marital status (n=204)	Single	146	(71.6)	-	-
	Married	58	(28.4)	-	-
Posttraumatic growth	Total score (0–132)	-	-	44.09	21.73
	New Possibilities (0–30)	-	-	10.19	5.53
	Personal strength(0–30)	-	-	9.17	4.73
	Relating to Others (0–42)	-	-	14.75	7.90
	Spiritual Change (0–12)	-	-	3.65	2.92
	Appreciation of life (0–18)	-	-	6.33	3.47
Most shocking incidents (n=200)	Physical abuse	75	(37.5)	-	-
	Emotional abuse	12	(6.0)	-	-
	Sexual abuse	52	(26.0)	-	-
	Neglect	61	(30.5)	-	-
Psychological pain	([1-10])	-	-	6.65	1.86
Posttraumatic stress	Total score (0–88)	-	-	28.73	20.05
	Invasion (0–32)	-	-	11.27	7.62
	Hyper-arousal (0–24)	-	-	6.67	6.07
	Avoidance (0–32)	-	-	10.78	7.50
Support in crises	(7–49)	-	-	29.7	7.72
Support from agencies (n=200)	None	174	(87.0)	-	-
	Vacation	3	(11.5)	-	-
	Job change	2	(7.7)	-	-
	Psychotherapy, Counseling	16	(61.5)	-	-
	Other^‡^	5	(23.1)	-	-
Colleagueship	Satisfaction	99	(48.5)	-	-
Help seeking (n=204)	Yes^§^	177	(86.8)	-	-
Experience of counseling (n=200)	Yes	13	(6.5)	-	-
Reason for not getting counseling (n=176)	Might be able to solve by myself	65	(36.9)	-	-
	Lack of information	12	(6.8)	-	-
	Lack of time	41	(23.2)	-	-
	Concern about others’ attention	7	(3.9)	-	-
	Cost	14	(7.9)	-	-
	Believe it does not help	22	(12.5)	-	-
	Other	15	(8.5)	-	-
Stress Coping	Total score (33–99)	-	-	61.71	10.68
	Ways of pursuing social support ([11-33])	-	-	21.16	4.56
	Problem solving-centered methods ([11-33])	-	-	23.70	5.19
	Avoidance-centered coping methods ([11-33])	-	-	16.78	4.27

^*^Sample sizes vary slightly because of missing data on some items.^†^Protestantism, Catholic, Buddhism, etc.

^‡^Duty change, Hospital care.

^§^To Collegue, Boss, Minister, Shaman, Doctor, Psychologist.

### Properties of major variables

Scores for PTG were 44.09 (SD=21.73) on average and the most shocking incidents of child abuse reported by the respondents were physical abuse 37.5% (75 persons), followed by non-intervention 30.5% (61 persons), sexual abuse 26% (52 persons) and emotional abuse 6% (12 persons). The figure related to the psychological pain caused by those incidents was 6.65 (SD=1.86) and 28.73 (SD=20.05) for posttraumatic stress, higher than PTSD selection cut off point of 24.

Scores for support in crises were 29.70 (SD=7.72) on average and it was found that the majority, or 87.0% (174 persons), couldn’t get support from agencies. Psychotherapy and counseling occupied the highest ratio of support from agencies at 61.53% (16 persons). Up to 177 persons (86.8%) have experienced vicarious trauma and sought help in any form for it, but just 13 persons (6.5%) had received counseling. As for the reasons for not getting counseling, 36.9% (65 persons) thought that they could solve problems by themselves and 23.2% (41 persons) replied that they had no time to do that. Regarding relations with colleagues and bosses, just 99 persons (48.5%) were satisfied, showing that more than half of the respondents were unsatisfied with relations with their co-workers and bosses.

Scores for social workers’ coping with stress were 61.71 (SD=10.68) on average. Scores for coping with stress by problem solving were highest in sub-scales with 23.70 (SD=5.19), followed by coping with stress by pursuing social support 21.16 (SD=4.56) (Table 1).

### Factors affecting posttraumatic growth

#### Univariate analysis

According to univariate analysis of factors affecting each area of PTG, religion affected spiritual change, and satisfaction with relationships with colleagues and supervisors affected total scores, relating to others, and new possibilities. Help-seeking affected total scores and all sub-scales except for spiritual change. Experience of counseling affected total scores. Total scores on Posttraumatic stress and all sub-scales affected total scores on the PTG and all sub-scales except for spiritual change. Support in crises affected total scores on the PTG and relating to others (Table 2–3).

**Table 2:** **The *t***-**test and ANOVA test for univariate analysis of factors influencing posttraumatic growth**^**1**)^

				**Subscales of Posttraumatic growth**														
	**Posttraumatic growth**			**Relating to others**			**New possibilities**			**Personal strength**			**Appreciation of life**			**Spiritual change**		
	**Mean**	**SD**	**t/F**	**Mean**	**SD**	**t/F**	**Mean**	**SD**	**t/F**	**Mean**	**SD**	**t/F**	**Mean**	**SD**	**t/F**	**Mean**	**SD**	**t/F**
Religion																		
Yes	--	--	-	--	--	-	--	--	-	--	--	-	--	--	-	4.17	2.88	−5.626^‡^
Non	--	--	-	--	--	-	--	--	-	--	--	-	--	--	-	1.36	1.74	−5.626^‡^
Colleagueship																		
Poor	38.70	22.13	4.667^*^	12.48	8.19	4.721^*^	9.30	6.09	3.356^*^	---	---	-	---	---	-	---	---	-
Moderate	40.01	21.81	4.667^*^	13.38	8.01	4.721^*^	9.21	5.38	3.356^*^	---	---	-	---	---	-	---	---	-
Satisfactory	48.78	20.77	4.667^*^	16.44	7.44	4.721^*^	11.21	5.36	3.356^*^	---	---	-	---	---	-	---	---	-
Help seeking																		
No	33.26	23.79	−2.275^*^	11.19	8.76	−2.907^*^	7.74	5.58	−2.458^*^	6.81	5.15	−2.583^*^	4.74	3.51	−2.583^*^	--	--	-
Yes	45.75	20.99	−2.275^*^	15.29	7.64	−2.907^*^	10.57	5.44	−2.458^*^	9.53	4.57	−2.583^*^	6.58	3.41	−2.583^*^	--	--	-
Experience of counseling																		
No	43.46	22.04	−2.408^*^	--	--	-	--	--	-	8.95	4.78	−2.217^*^	--	--	-	--	--	-
Yes	54.85	16.30	−2.408^*^	--	--	-	--	--	-	11.92	2.47	−2.217^*^	--	--	-	--	--	-

^1)^ Only statistically significant variables are presented in the table. t-tests were used for Sex, Education, Religion, Marital status, Most shocking incidents, Colleagueship satisfaction, Help seeking, and Experience of counseling, One-way ANOVA was used for age, Most shocking incidents, Support from agencies, and Reason for not getting counseling.

^*^p<0.05, ^†^p<0.01, ^‡^p<0.001.

**Table 3:** **Univariate regression for analysis of factors influencing posttraumatic growth**^**1**)^

			**Subscales of Posttraumatic growth**									
	**Posttraumatic growth**		**Relating to others**		**New possibilities**		**Personal strength**		**Appreciation of life**		**Spiritual change**	
	**B**	**Beta**	**B**	**Beta**	**B**	**Beta**	**B**	**Beta**	**B**	**Beta**	**B**	**Beta**
Psychological pain	0.245^‡^	3.589	0.257^‡^	3.775	0.203^†^	2.952	0.228^†^	3.327	0.188^†^	2.727	0.150^*^	2.152
Posttraumatic stress												
Hyper-arousal	0.260^‡^	3.824	0.225^†^	3.279	0.273^‡^	4.030	0.196^†^	2.835	0.334^‡^	5.034	--	---
Invasion	0.314^‡^	4.706	0.276^‡^	4.082	0.325^‡^	4.891	0.255^‡^	3.746	0.373^‡^	5.715	---	---
Avoidance	0.290^‡^	4.312	0.256^‡^	3.757	0.278^‡^	4.108	0.255^‡^	3.742	0.342^‡^	5.167	---	---
Support in crises	0.145^*^	2.072	0.148^*^	2.117	-	-	-	-	-	-	-	-
Stress Coping												
Ways of pursuing social support	0.482^‡^	7.778	0.486^‡^	7.862	0.424^‡^	6.621	0.442^‡^	6.966	0.360^‡^	5.464	0.331^‡^	4.961
Problem solving-centered methods	0.271^‡^	3.957	0.245^‡^	3.558	0.266^‡^	3.886	0.288^‡^	4.226	0.232^†^	3.349	0.110	1.558
Avoidance-centered coping methods	0.339^‡^	5.101	0.333^‡^	5.002	0.309^‡^	4.595	0.266^‡^	3.895	0.271^‡^	3.982	0.290^‡^	4.288

^1)^ Only statistically significant variables are presented in the table. Regression was used for Psychological pain, Posttraumatic stress (Invasion, Hyper-arousal, Avoidance), Support in crises, Stress Coping (Ways of pursuing social support, Problem solving-centered methods, Avoidance-centered coping methods).

^*^p<0.05, ^†^p<0.01, ^‡^p<0.001.

### Correlation among independent variables

The correlationship among variables was analyzed to confirm multi-collinearity between independent variables before conducting regression analysis. If the value of a coefficient of correlation is 0.80 or higher, it can be said that multi-collinearity exists between the variables. In this study, the correlation among the sub-scales of invasion, hyper-arousal, and avoidance was 0.80 or higher, indicating that there was multi-collinearity. In the hierarchical multiple regression model, hyper-arousal and avoidance were excluded for the explanatory power of the model. All correlation coefficients between other variables were 0.80 or lower, tolerance limits were less than 1, and the VIF(varience inflation factor) was 2.5 or lower, showing that there was not multi-collinearity ploblem among independent variables. The value of Durbin-Watson was 1.677, close to the value of 2, and was not close to 0 or 4, indicating that there were no multi-collinearity between residuals, and thus a hierarchical linear modeling was conducted (Table 4).

**Table 4:** Correlation among independent variables

		**1**	**2**	**3**	**4**	**5**	**6**	**7**	**8**	**9**	**10**	**11**	**12**	**13**
Sociodemographic														
1	Religiousness	1												
2	Age: 20s	0.003	1											
3	Psychological pain	−0.028	−0.116	1										
Posttraumatic stress														
4	Invasion	−0.006	0.059	0.439^‡^	1									
5	Hyper-arousal	−0.061	0.092	0.350^‡^	0.886^‡^	1								
6	Avoidance	−0.023	0.053	0.393^‡^	0.825^‡^	0.817^‡^	1							
Social support														
7	Support in crises	0.027	−0.047	−0.057	−0.104	−0.121	−0.079	1						
8	Colleagueship	0.048	0.094	0.020	−0.037	−0.109	−0.034	0.234^†^	1					
9	Help seeking	−0.062	0.055	0.082	0.172^*^	0.165^*^	0.198^†^	0.254^‡^	0.135	1				
10	Experience of counseling	0.063	−0.030	0.087	0.053	0.042	0.052	−0.066	0.042	0.104	1			
Stress Coping														
11	Ways of pursuing social support	0.039	0.053	0.257^‡^	0.386^‡^	0.307^‡^	0.306^‡^	0.222†	0.136	0.401^‡^	0.069	1		
12	Problem solving-centered methods	−0.027	−0.118	0.198^†^	0.224^†^	0.188^†^	0.148^*^	0.173^*^	0.070	0.109	0.064	0.546^‡^	1	
13	Avoidance-centered coping methods	0.070	0.017	0.272^‡^	0.564^‡^	0.526^‡^	0.580^‡^	0.003	0.009	0.202^†^	0.021	0.444^‡^	0.105	1

^*^p<0.05, ^†^p<0.01, ^‡^p<0.001.

### Factors affecting Posttraumatic Growth: Hierarchical multiple regression

According to an analysis of influence of sociodemographic variables included in the first stage, the model was not statistically significant (F=1.640, p<0.05) and explanatory power was as low as 1.7%, showing that there was no independent influence of sociodemographic characteristics. The explanatory power of the second stage including trauma-related variables was 13.1%, which was 11.4% higher than the explanatory power with just the sociodemographic variables, and the model was statistically significant (F=7.040, p<0.001). In the second stage, religion and invasion were variables significantly affecting PTG. Higher scores with regard to invasion (β=0.256, p<0.01) meant higher PTG. In the third stage, the explanatory power of a model with the addition of social support variables increased 8.1% to 21.2% (F=6.150, p<0.001). The invasion factor (β=0.257, p<0.01) and satisfaction with relationships with colleagues and supervisors (β=0.146, p<0.05) had significant effects on PTG. In a model of the final fourth stage with the addition of the stress coping factor, the PTG of social workers at child protective agencies was 31.9%, showing that the explanatory power increased 10.7% from Model 3. The factors of invasion and relationships with colleagues and supervisors, which were significant in the previous models, were not statistically significant in Model 4 with the stress coping factor added. With the stress coping factor, the PTG was higher when using the social support pursuit method (β=0.319, p<0.001) (Table 5).

**Table 5:** Multivariate analysis of factors influencing posttraumatic growth

**Model**	**1**		**2**		**3**		**4**	
	**B**	**β**	**B**	**β**	**B**	**β**	**B**	**β**
Sociodemographic								
Religiousness	7.551	0.128	7.707	0.131	6.746	0.114	5.477	0.093
Age: 20s	1.351	0.030	1.291	0.029	0.282	0.006	0.275	0.006
Psychological pain			1.586	0.132	1.564	0.130	1.120	0.093
Invasion			0.729^†^	0.256	0.734^†^	0.257	0.255	0.089
Social Support								
Support in crises					0.371	0.130	0.178	0.062
Colleagueship					4.521^*^	0.146	3.706	0.119
Help seeking					7.387	0.109	1.241	0.018
Experience of counseling					8.077	0.092	7.391	0.084
Stress Coping								
Ways of pursuing social support							1.580^‡^	0.319
Problem solving-centered methods							0.196	0.046
Avoidance-centered coping methods							0.560	0.109
**Constant**	36.906^‡^		17.958^*^		−4.614		−30.533^†^	
**R2**	0.017		0.131		0.212		0.319	
**Adjusted R2**	0.007		0.112		0.177		0.277	
**F**	1.640		7.040^‡^		6.150^‡^		7.669^‡^	
**R**^**2 **^**Change**			0.114		0.081		0.107	

^*^p<0.05, ^†^p<0.01, ^‡^p<0.001.

## Discussion

This study investigated the PTG that was experienced by social workers at child protective agencies and analyzed sociodemographic characteristics, vicarious trauma-related factors, social support-related factors, and stress coping methods in order to find factors relevant to PTG.

It should first be noted that the total score for PTG was 44.09 (SD=21.73), relatively lower than the score of 53.7 (SD=11.8) reported by a study on social workers handling trauma conducted by Gibbons et al. (2011) [16] using almost the same tools and 64.42 (SD=20.08) by a study on therapists conducted by Linley and Joseph (2007) [18]. However, it is difficult to generalize about the lower PTG of Korean social workers at child protective agencies compared to non-Koreans with similar jobs and follow-up studies are needed for verification. If the PTG is low despite the similarity in jobs, it is necessary to clarify factors regarding working conditions.

Second, according to an analysis of vicarious trauma-related factors, the score for posttraumatic stress was as high as 28.73 (SD=20.05) on average, higher than 24 points, which is the cut-off score for PTSD selection. Such a result agrees with the results of Korean and international studies indicating that the risk of posttraumatic stress disorder was remarkably high among persons providing child protective services [6,7,27,28]. Posttraumatic stress from work not only causes depression, uneasiness, impulsive acts, drug abuse, and somatization, but can also have harmful effects on organizations and lower the quality and efficiency of work [29,30]. Therefore, vicarious trauma experienced by social workers should be addressed and protective measures should be taken. The degree of those social workers’ vicarious trauma should be regularly evaluated to determine if they belong to a risk group and intensive mental health services should be provided if necessary. Psychological anguish was included in the study design stage on the assumption that PTG appears as a result of the cognitive processing that takes place to deal with psychological anguish after vicarious trauma [9,11]. It was hard to compare the results of this study with single item tool from other existing studies. In follow-up studies, more sophisticated tools which can measure the persons experiencing vicarious trauma should be used.

Third, in terms of social support, social workers providing child protective services did try to seek help from surrounding people regarding their vicarious trauma, but the ratio of accessing professional support, such as psychotherapy, was low. Considering that about 86.8% of respondents have sought help, they did not seem to be familiar with undergoing counseling, even if they felt the need for counseling. In fact, there have been reports that persons influenced by Asian cultural attitudes and norms are not familiar or comfortable with counseling because of various aspects of Asian culture, such as attitudes toward authority or the emphasis on hierarchical relationships [31]. It is necessary for organizations to actively provide counseling channels and educational opportunities that workers can easily access, instead of letting individuals look for channels to obtain help.

Fourth, regarding stress coping scores, the preferred coping method of social workers at child protective agencies was problem solving. The least frequently used method was avoidance.

Fifth, in the univariate analysis of factors related to PTG, scores for PTG were higher when respondents had religious beliefs, when they maintained friendly relationships with colleagues and supervisors, when they sought help, when they received counseling, when they had serious psychological anguish, when they experienced serious posttraumatic stress, including hyper-arousal, invasion, and avoidance, and when they actively attempted to cope with stress. The relationship between PTG and religion supports the results of existing studies [18,32] and also correlates with the results of studies conducted by Prati and Pietrantoni (2009) [19] and Schaefer and Moos (1998) [33] that social support affects PTG. Tedeschi and Calhoun (2004) [9], who suggested the concept of PTG, also stated that social support was one of the most important predictors of positive changes after trauma. The results concerning trauma-related factors agree with those of many studies that have clarified the positive relationship between trauma stress and PTG [25,32]. Serious post-traumatic stress, even if the stress is painful, has been shown through the results of various studies to promote post-traumatic growth as a result of reflecting on the trauma experience. Through such reflection, new meanings are created and the collapsed world is rebuilt [13,34].

Hierarchical regression was conducted to analyze the final model in order to look for factors affecting PTG. In the final model, the effects of other factors were offset when the four-stage stress coping factors were included and the pursuit of social support seemed to be the most powerful factor. Schaefer and Moos (1998) [33] and Shakespeare-Finch et al. (2005) [35] have noted that active coping actions with regard to life crises result in adaptation and personal growth. In a meta-analysis of the results of factors affecting PTG, Prati and Pietrantoni (2009) [19] stated that, out of the different coping strategies, the coping method of looking for social support was one of the most important factors affecting PTG. Religion, social support, and factors related to the degree of trauma which were meaningful in the univariate analysis were offset in the final model when coping strategies were taken into consideration. Such a result shows that personal factors or the degree of shock that persons experience from incidents, stress, and social support certainly are important for social workers, but it was found that their coping strategies with regard to crises, and active strategies for looking for social support systems to solve problems in particular, were important factors in achieving growth despite trauma or stress.

This study has limitations. First, it is difficult to prove causal relationships between each independent variable and dependent variable because of the properties of cross-sectional studies. Second, regarding variables, such as psychological anguish and support during crises, this study was designed to recollect the experiences related to the most shocking incidents, but the accuracy of recollection can vary because respondents experienced the most shocking incidents at different times. Third, regarding post-traumatic stress, this study was designed to measure the present stress, but the level of stress could have been affected by when respondents experienced those incidents. Fourth, regarding psychological anguish, this study used a single item tool so that respondents could talk about the level of psychological anguish immediately after the most shocking incident on the assumption that post-traumatic growth appeared in the process of handling anguish based on previous studies. More sophisticated tools should be included in follow up studies. In spite of all these limitations, this study examined, for the first time in Korea, the factors affecting posttraumatic stress among social workers at child protective agencies that care for child victims of violence.

Social workers at child protective agencies perform very important duties but their mental health is vulnerable due to vicarious trauma and their turnover rate is very high. As a result, problems arise related to the connectivity and continuity of child protective services. Attention should be paid to mental health issues related to vicarious trauma, in order to protect these social workers and to help them carry on with their jobs, on which the quality of child protective services depends. In addition, efforts should be made to promote post-traumatic growth, which can work as a protective factor. In this study it was found that using coping methods that involved pursuing social support had significant effects on PTG. Therefore, it is suggested that a study be developed that focuses on how to activate stress coping behavior by methods that involve pursuing social support.

## Competing interests

The authors declare that they have no competing interests.

## Authors’ contributions

Han IY designed first research model and supported fund for study. All authors developed research model, especially Go YB collected data and Rhee, YS analyzed the statistics and wrote the manuscript. All authors read and approved the final manuscript.
